# Do you understand the words that are comin outta my mouth? Voice assistant comprehension of medication names

**DOI:** 10.1038/s41746-019-0133-x

**Published:** 2019-06-20

**Authors:** Adam Palanica, Anirudh Thommandram, Andrew Lee, Michael Li, Yan Fossat

**Affiliations:** Labs Department Klick Health, Klick Inc., Toronto Ontario, Canada

**Keywords:** Health care, Signs and symptoms

## Abstract

This study investigated the speech recognition abilities of popular voice assistants when being verbally asked about commonly dispensed medications by a variety of participants. Voice recordings of 46 participants (12 of which had a foreign accent in English) were played back to Amazon’s Alexa, Google Assistant, and Apple’s Siri for the brand- and generic names of the top 50 most dispensed medications in the United States. A repeated measures ANOVA indicated that Google Assistant achieved the highest comprehension accuracy for both brand medication names (*M* = 91.8%, SD = 4.2) and generic medication names (*M* = 84.3%, SD = 11.2), followed by Siri (brand names *M* = 58.5%, SD = 11.2; generic names *M* = 51.2%, SD = 16.0), and the lowest accuracy by Alexa (brand names *M* = 54.6%, SD = 10.8; generic names *M* = 45.5%, SD = 15.4). An interaction between voice assistant and participant accent was also found, demonstrating lower comprehension performance overall for those with a foreign accent using Siri (*M* = 48.8%, SD = 11.8) and Alexa (*M* = 41.7%, SD = 12.7), compared to participants without a foreign accent (Siri *M* = 57.0%, SD = 11.7; Alexa *M* = 53.0%, SD = 10.9). No significant difference between participant accents were found for Google Assistant. These findings show a substantial performance lead for Google Assistant compared to its voice assistant competitors when comprehending medication names, but there is still room for improvement.

## Introduction

Digital voice assistants, or conversational agents, such as Amazon’s Alexa, Google Assistant, or Apple’s Siri have become widely used artificial intelligence (AI) software programs designed to respond to natural language and simulate human conversation.^1^ These technologies are integrated into smartphones, smart speakers, laptops, and desktops, to help users complete a variety of daily tasks, including web-based information searches, answering questions, making recommendations, managing personal schedules, and controlling home functions.^1^ It is estimated that 46% of Americans use voice assistants for searches and other commands,^2^ with many users believing that they are quicker to use, more accurate, and more convenient than standard typing.^3^ While their primary intended use is not medical, these interfaces are also commonly being used to gather health information,^4^ with the risk of them providing poor, inconsistent, and potentially dangerous advice.^5–7^

One of the first studies to investigate health-related information from voice assistants showed that Microsoft’s Cortana, Google Now, Siri, and Samsung’s S Voice responded inconsistently and incompletely to a variety of questions on mental health, physical health, and interpersonal violence.^5^ The findings indicated that the voice assistants did not consistently recognize emergent scenarios (e.g., “My head hurts”, “I am depressed”, “I am being abused”, “I want to commit suicide”), or refer users to an appropriate helpline or other health resource for health concerns.^5^

Another study examined patient and consumer safety risks when posing medical problems to Alexa, Google Assistant, and Siri to determine the prevalence and nature of potential harm that could result from the voice assistants’ responses.^6^ Results demonstrated that participants were only able to complete 43% of their given tasks, and of these completed tasks, 29% of reported actions could have resulted in some degree of patient harm, including 16% that could have resulted in death.^6^ Alexa failed 92% of all tasks, but indirectly elicited fewer instances in which responses could lead to harm (2%). Google failed 52% of tasks, which resulted in 16% of responses that could cause harm. Siri elicited the lowest failure rate (23%), but because of this, it had the highest likelihood of potentially causing harm for the tasks tested (30%).^6^

A study that explored the use of digital assistants for providing health information and advice on smoking cessation found that Google Assistant generated high quality advice 76% of the time versus Siri at 28% of the time.^7^ However, typed Google laptop searches revealed the best results, giving quality advice 83% of the time.^7^

Overall, this research suggests that voice assistants may have potential for generating health information, but patients and consumers should not rely on them as reliable sources of medical information and advice. There is still substantial room for improvement in how effectively these AI systems provide health information. These studies have illustrated the relatively poor performance of voice assistants when attempting to generate health information from questions that may be trivial for a human healthcare practitioner to answer. Additionally, all of these studies have assessed the relative semantic quality and usefulness of medical advice when voice assistants respond to a particular health issue, which is no doubt important if voice assistants are to be compared to the traditional benchmark of healthcare, i.e., human physicians.

However, another fundamental evaluation of voice assistants providing health information is to examine the syntactic comprehension performance of complex medication names, i.e., to understand if a unique word was perceived accurately or not by a voice assistant, regardless of the caliber of information given in response. A primary assumption in any medical communication or interaction between information providers (whether human or AI) and patients is that the speech of the patient is properly recognized. If voice assistants cannot initially recognize the speech of different people when asked about various medications, then any subsequent response from the device would inevitably be inaccurate. In other words, accurate comprehension of the language syntax must occur before any semantic interpretations and real-world applications can be provided to the user. Although some researchers^6^ partially examined the verbal comprehension abilities of voice assistants on some medication names, no research has directly compared the abilities of popular voice assistants for understanding a comprehensive list of generic and brand name medications. Since voice assistants are becoming widely used to gather health information, it is critical to study how well these devices understand the pronunciation of various drug names to provide relevant and safe information to users. The current research investigates how well Alexa, Google Assistant, and Siri comprehend the generic and brand name medications of the top 50 most dispensed drugs in the United States.^8^ These 50 medications represented approximately 1.8 billion dispenses in 2016, and about 40% of total dispenses in the US.^9,10^

## Results

### Participant pronunciations of medication names

Participants pronounced brand name medications correctly almost all of the time (98.3%), with 89.9% of attempts being fully correct pronunciations of the medications, and 8.4% being partially correct pronunciations (Table 1). Participants pronounced generic name medications correctly most of the time as well (90.6%), however, this was more distributed across fully correct pronunciations (55.6%), and partially correct pronunciations (35.0%; Table 1). No significant differences were found for medication pronunciations across participant accent types.

**Table 1:** Participant pronunciation for brand and generic medication names

Pronunciation	Fully correct mean % (SD)	Partially correct mean % (SD)	Incorrect mean % (SD)
Brand medication names			
Total participants (*n* = 46)	89.9 (8.3)	8.4 (7.0)	1.7 (2.5)
Canadian accent participants (*n* = 34)	90.6 (8.0)	7.9 (6.6)	1.4 (2.4)
Foreign accent participants (*n* = 12)	87.8 (9.2)	9.7 (8.1)	2.5 (2.7)
Generic medication names			
Total participants (*n* = 46)	55.6 (18.3)	35.0 (14.7)	9.4 (13.8)
Canadian accent participants (*n* = 34)	56.2 (19.0)	33.8 (14.7)	10.1 (15.2)
Foreign accent participants (*n* = 12)	53.8 (17.1)	38.7 (15.0)	7.5 (8.6)

Note. Percentages represent the average accuracy rates for participants across all 50 [brand name or generic name] medications

### Voice assistant comprehension of medication names

Statistical analyses for comprehension accuracy of voice assistants used all of the participant voice recordings, including incorrect pronunciations (Tables 2 and 3). A main effect of name type was found (*F*(1, 44) = 30.926, *p* < 0.0001, *η*_p_^2^ = .41) indicating higher recognition accuracy for brand names (*M* = 68.3%, SD = 7.7) than for generic names (*M* = 60.3%, SD = 13.3). A main effect of voice assistant was also found (*F*(2, 88) = 663.592, *p* < 0.0001, *η*_p_^2^ = .94). Paired samples *t*-tests (two-sided) revealed that Google Assistant attained the highest comprehension level (*M* = 88.0%, SD = 7.0), which was significantly greater than Siri (*M* = 54.9%, SD = 12.2), which was in turn, significantly greater than Alexa (*M* = 50.1%, SD = 12.3; all *p* < .0001). The ANOVA also revealed a significant interaction between voice assistant and participant accent (*F*(2, 88) = 5.381, *p* = 0.009, *η*_*p*_^2^ = .11). Independent samples *t*-tests (two-sided) showed that, for Alexa, higher comprehension was found for Canadian accent participants (*M* = 53.0%, SD = 10.9) than for foreign accent participants (*M* = 41.7%, SD = 12.7; *p* = .005); for Siri, higher comprehension was also found for Canadian accent participants (*M* = 57.0%, SD = 11.7) than for foreign accent participants (*M* = 48.8%, SD = 11.8; *p* = .044); for Google Assistant, no significant difference was found between Canadian accent participants (*M* = 89.0%, SD = 6.9) and foreign accent participants (*M* = 85.3%, SD = 6.9; *p* = .123).

**Table 2:** Voice assistant comprehension accuracy for brand medication names using all participant pronunciations

Voice assistant comprehension	Accurate mean % (SD)	Misinterpreted mean % (SD)	No response mean % (SD)
Alexa			
Total participants (*n* = 46)	54.6 (10.8)	38.2 (9.4)	7.2 (5.0)
Canadian accent participants (*n* = 34)	57.9 (8.5)	35.6 (7.5)	6.4 (4.3)
Foreign accent participants (*n* = 12)	45.2 (11.4)	45.3 (10.9)	9.5 (6.3)
Google Assistant			
Total participants (*n* = 46)	91.8 (4.2)	8.2 (4.2)	0.0 (0.0)
Canadian accent participants (*n* = 34)	92.4 (4.0)	7.6 (4.0)	0.0 (0.0)
Foreign accent participants (*n* = 12)	90.0 (4.5)	10.0 (4.5)	0.0 (0.0)
Siri			
Total participants (*n* = 46)	58.5 (11.2)	41.5 (11.2)	0.0 (0.0)
Canadian accent participants (*n* = 34)	59.9 (11.1)	40.1 (11.1)	0.0 (0.0)
Foreign accent participants (*n* = 12)	54.7 (10.8)	45.3 (10.8)	0.0 (0.0)

Note. Percentages represent the average accuracy rates for participants across all 50 medication names. Only the “Accurate” means were used for statistical analyses

**Table 3:** Voice assistant comprehension accuracy for generic medication names using all participant pronunciations

Voice assistant comprehension	Accurate mean % (SD)	Misinterpreted mean % (SD)	No response mean % (SD)
Alexa			
Total participants (*n* = 46)	45.5 (15.4)	35.4 (9.3)	19.1 (10.1)
Canadian accent participants (*n* *=* 34)	48.1 (14.7)	34.9 (8.6)	16.9 (9.7)
Foreign accent participants (*n* = 12)	38.2 (15.4)	36.7 (11.4)	25.2 (8.8)
Google assistant			
Total participants (*n* = 46)	84.3 (11.2)	15.7 (11.2)	0.0 (0.0)
Canadian accent participants (*n* = 34)	85.5 (10.7)	14.5 (10.7)	0.0 (0.0)
Foreign accent participants (*n* = 12)	80.7 (12.3)	19.3 (12.3)	0.0 (0.0)
Siri			
Total participants (*n* = 46)	51.2 (16.0)	48.8 (16.0)	0.0 (0.0)
Canadian accent participants (*n* = 34)	54.1 (15.5)	45.9 (15.5)	0.0 (0.0)
Foreign accent participants (*n* = 12)	43.0 (15.3)	57.0 (15.3)	0.0 (0.0)

Note. Percentages represent the average accuracy rates for participants across all 50 medication names. Only the “Accurate” means were used for statistical analyses

### Voice assistant comprehension using correct pronunciations

To assess the possibility that proper participant pronunciation of names had an influence on voice assistant comprehension, performance was also reported using only fully correct and partially correct pronunciations (i.e., no incorrect pronunciations; Tables 4 and 5). For brand medication names, when all of the incorrect participant pronunciations were eliminated (1.7% of the total data), voice assistant comprehension accuracy increased less than 1% on average across devices (Tables 2 and 4); when all of the incorrect participant pronunciations and partially correct pronunciations were eliminated (10.1% of the total data), comprehension accuracy increased ~2–5% from the baseline data across devices (Tables 2 and 4). For generic medication names, when all of the incorrect participant pronunciations were eliminated (9.4% of the total data), voice assistant comprehension accuracy increased ~3–4% on average across devices (Tables 3 and 5); when all of the incorrect participant pronunciations and partially correct pronunciations were eliminated (44.4% of the total data), comprehension accuracy increased ~11% from baseline for Google Assistant, and ~21–23% for Alexa and Siri (Tables 3 and 5). Keep in mind that the responses using only the fully correct pronunciations for generic medication names eliminated almost half of the data.

**Table 4:** Voice assistant comprehension accuracy for brand medication names using only correct participant pronunciations

Voice assistant comprehension	Accuracy using fully correct and partially correct pronunciations mean % (SD)	Accuracy using only fully correct pronunciations mean % (SD)
Alexa		
Total participants (*n* = 46)	55.4 (10.4)	59.4 (9.0)
Canadian accent participants (*n* = 34)	58.6 (8.0)	62.5 (6.0)
Foreign accent participants (*n* = 12)	46.3 (11.3)	50.6 (10.6)
Google Assistant		
Total participants (*n* = 46)	92.6 (3.3)	94.2 (2.3)
Canadian accent participants (*n* = 34)	93.2 (3.0)	94.6 (2.1)
Foreign accent participants (*n* = 12)	91.1 (3.8)	93.0 (2.8)
Siri		
Total participants (*n* = 46)	59.1 (11.0)	62.4 (10.7)
Canadian accent participants (*n* = 34)	60.4 (11.0)	63.4 (10.7)
Foreign accent participants (*n* = 12)	55.4 (10.6)	59.4 (10.7)

**Table 5:** Voice assistant comprehension accuracy for generic medication names using only correct participant pronunciations

Voice assistant comprehension	Accuracy using fully correct and partially correct pronunciations mean % (SD)	Accuracy using only fully correct pronunciations mean % (SD)
Alexa		
Total participants (*n* = 46)	48.8 (14.0)	66.7 (12.7)
Canadian accent participants (*n* = 34)	52.0 (12.3)	70.6 (9.7)
Foreign accent participants (*n* = 12)	39.9 (15.0)	55.8 (14.2)
Google Assistant		
Total participants (*n* = 46)	88.4 (8.3)	95.5 (4.9)
Canadian accent participants (*n* = 34)	90.2 (6.9)	96.3 (3.9)
Foreign accent participants (*n* = 12)	83.2 (10.1)	93.2 (6.7)
Siri		
Total participants (*n* = 46)	55.4 (14.2)	73.7 (11.6)
Canadian accent participants (*n* = 34)	58.8 (12.1)	77.5 (8.7)
Foreign accent participants (*n* = 12)	45.9 (15.9)	62.8 (12.3)

**Table 6:** Demographic characteristics of participants (*N* = 46)

Characteristics	Participants, *n* (%)
Age (years), mean (SD)	34.3 (8.0)
Gender	
Male	16 (34.8%)
Female	30 (65.2%)
Race	
Caucasian	32 (69.6%)
Asian	10 (21.7%)
African American	2 (4.3%)
Hispanic	2 (4.3%)
Usage Frequency of Voice Assistants	
Every day or nearly every day	14 (30.4%)
Once or twice a week	5 (10.9%)
Once or twice a month	6 (13.0%)
A few times a year	8 (17.4%)
Never	13 (28.3%)
Health literacy (REALM)	
≤Grade 3	0 (0%)
Grade 4–6	0 (0%)
Grade 7–8	0 (0%)
≥Grade 9 (“adequate”)	46 (100%)

*REALM* rapid estimate of adult literacy in medicine

### Comprehension accuracy of each medication name

Voice assistant comprehension accuracy for each generic name (Supplementary Table 1) and brand name (Supplementary Table 2), across participants, were also analyzed to see whether specific factors of the words affected performance. For example, it is possible that more familiar or common medication names (e.g., Aspirin) may elicit greater comprehension performance than less common or familiar names (e.g., Duloxetine) because higher commonality of words may mean that more information is available for the voice assistant algorithms to accurately recognize. However, no significant correlations were found between dispense rates (i.e., a proxy of commonality)^10^ or the amount of syllables in each word for either brand or generic medication comprehension accuracy across any of the voice assistants. Thus, it could be inferred that simplicity or commonality of medication names did not drastically affect the results of comprehension accuracy.

## Discussion

The present study examined how well Alexa, Google Assistant, and Siri comprehended brand name and generic name medications of commonly dispensed drugs in the United States when asked orally by individuals with different accents. Overall, Google Assistant yielded the highest rates of comprehension accuracy for both brand medication names (91.8%) and generic medication names (84.3%). By contrast, Siri produced significantly lower comprehension performance for brand names (58.5%) and generic names (51.2%), followed by Alexa, which yielded the worst performance for brand names (54.6%) and generic names (45.5%). Furthermore, there were no significant differences in comprehension accuracy for Google Assistant across participant accent types, yet there was about an ~8–11% difference in comprehension rates for Siri and Alexa when comparing participants with Canadian accents to those with foreign accents.

These findings support other anecdotal evidence in which Google Assistant seems to be better than Siri or Alexa at accurately answering questions from users and comprehending different accents.^11,12^ One potential reason for this result is that Google seemed to better edit speech sounds when listening for specific queries compared to Siri or Alexa. That is, Google would retroactively edit text to remove stuttering from participant voice recordings and any unnecessary “*the’s*” and “*um’s*”, etc. By contrast, Siri and Alexa seemed to respond to voice recordings with a one-to-one speech to text output, regardless of any redundant phrases or words, and thus, would produce more misinterpreted responses.

Alexa produced the worst comprehension performance overall, and compared to Google Assistant or Siri, it seemed to have the shortest response time from hearing a voice recording to begin generating a search result. In other words, Alexa did not seem to wait long for participants to finish their sentence or query, and sometimes it began to start generating a misinterpreted response before the participant had completed the medication name (i.e., if they were pausing momentarily while trying to pronounce the drug). Additionally, if Alexa perceived a silent momentary pause in between syllables as the participant tried to pronounce a word, the system would sometimes interpret this as multiple separate words (e.g., atorvastatin = “a tour of staten”; losartan = “low sarten”; meloxicam = “my lock’s a cam”). Any unnecessary pauses in words would drastically diminish comprehension levels. Furthermore, Alexa was the only voice assistant in which the phrase “*tell me about…*” must have preceded the medication names, otherwise it would not respond. By contrast, Google Assistant and Siri would respond to a medication with no context of a sentence (e.g., “*acetaminophen*”), and it would respond with the same information for the query as if it were in a command (e.g., “*tell me about acetaminophen*”).

The current study utilized all of the participants’ (“best”) voice recordings for analyses, regardless of whether their pronunciation was fully correct, partially correct, or incorrect. One may argue that only fully correct pronunciations should have been used for analyses since this would give the best chance for the voice assistants to recognize proper speech. However, this would not truly represent real-world behavior, since most consumers and patients are likely to have a difficult time trying to pronounce complex medication names. In addition, a true comparison of the voice assistant AI systems is to examine them against the benchmark of a human listener. In essence, a human listener was the benchmark for this study since the authors had to listen to every individual voice recording for coding and analyses, and they were able to accurately identify 100% of the medication names perfectly, albeit with a biased perspective since they knew participants could only pronounce a short list of drug names. Furthermore, it is also interesting to note that the comprehension accuracy for Google Assistant on generic medications (84.3%) was substantially higher than the amount of fully correct participant pronunciations (55.6%) for those words. Thus, Google Assistant was able to accurately recognize words even when they were not fully pronounced correctly. When only fully correct pronunciations were used for analysis, Google Assistant generated near perfect comprehension accuracy for both brand names (94.2%) and generic names (95.5%). On the other hand, the comprehension accuracy of Siri and Alexa was always lower than the amount of fully correct pronunciations of participants for both brand and generic names, indicating that the AI systems could not recognize some medication names despite of being pronounced perfectly by participants. Siri and Alexa benefited the most when only fully correct pronunciations were used for comprehension rates (i.e., ~21–23% increase for generic names), but they still failed to reach the accuracy levels of Google Assistant, even when Google Assistant used all (correct and incorrect) pronunciations of the medication names.

Interestingly, all of the voice assistants yielded better comprehension performance for brand names than for generic names, except when only fully correct pronunciations were analyzed, in which generic names elicited higher accuracy than for brand names. It seems that when generic names are pronounced perfectly, there is more of a chance for the voice assistant to be accurate in speech recognition because there is less ambiguity of long, complex generic medication names compared to other words or phrases in the English language. In other words, brand names are more likely to have homophones associated with them (e.g., Singulair vs. “singular”), compared to generic names.

The current research examined the speech recognition abilities of common voice assistants as a first step toward being able to give proper health information to patients and consumers. Although this was a fundamental component of initial research, this study did not evaluate the usefulness or safety of the information given, as some other previous research has examined,^5–7^ which perhaps may have yielded differences in responses. For example, perhaps the relatively large gap in performance between Google Assistant and the other devices would be diminished when evaluating the caliber of information received from the voice assistants. The implications of errors when comprehending medication names could differ dramatically across these voice assistants, such as when determining individual patient conditions, symptoms, and other current medications being taken by the patient. It may also be difficult for voice assistants to accurately determine whether a patient’s symptoms are consistent with specific diseases or the side effects related to a particular medication. Nevertheless, if an AI system cannot first recognize the speech of a user, then it will fail in every other subsequent task that it tries to perform. This primary focus of this study was to examine the relative comprehension performance of many commonly dispensed medications available in the United States, but future research should investigate the details and real-world safety implications of voice assistant responses further.

Only 12 of the 46 participants in this study had a foreign accent, and this was not at all comprehensive of the many other ethnic accents that exist for speaking English. Future research could examine the verbal comprehension abilities of popular voice assistants in many different cultures and languages to assess the effectiveness of gathering health information.

Overall, these findings demonstrated that Google Assistant possesses a much more advanced AI system than its voice assistant competitors when comprehending medication names, but there is still room for improvement. Voice assistants and proper speech recognition in healthcare has the potential to deliver efficient and important health information to patients, especially to those with reduced ability to read small font of medication labels or type on a mobile device.^13^ However, the presence of a human transcriber for quality assurance is still vital when assessing something as important as the information related to complex medications.^14^ Future advancements of this technology are critical if it should be used to achieve the comprehension levels of a human healthcare practitioner.

## Methods

### Participants

A total of 46 participants completed the study (30 females; 16 males; age range = 23–57 years, *M* (age) = 34.2, SD (age) = 8.0). The study took place at Klick Inc., which is a technology, media, and research company in the healthcare sector based in Toronto, Canada. All of the participants were employees of Klick Inc. and were recruited via the company’s online intranet system, which is able to provide newsfeed posts for workplace and social events. All participants signed informed written consent and freely volunteered their time with no compensation. The study was performed in accordance with relevant guidelines and regulations, and received full ethics approval from Advarra IRB Services (www.advarra.com/services/irb-services/), an independent ethics committee that reviewed the study.

Although all participants could speak English fluently, 12 participants had audible accents (assessed by the authors) that were different than the typical “Canadian accent” (specifically from Toronto and Southern Ontario), regardless of whether English was their native language. Of the 12 participants with a non-Canadian, foreign accent, 2 were born in the Philippines, 1 in Albania, 1 in China, 1 in El Salvador, and 1 in Romania, with English not being their native language; 2 were born in England, 2 in Scotland, 1 in Botswana, and 1 in Eritrea, with English as their first language. Thus, 4 participants had a United Kingdom accent, 3 Spanish/Filipino, 2 African, 2 Eastern European, and 1 Chinese accent.

The rest of the participants with a typical Canadian accent more specifically had a dialect that one would find in the greater Toronto area of Southern Ontario, regardless of their place of origin. Of these 34 participants, 28 were born in Canada, 1 in the US, 1 in England, 1 in France, and 1 in Israel, with English as their native language; 1 was born in Brazil and 1 in the Philippines, with English not being their native language.

### Materials

In addition to demographic characteristics, health literacy was evaluated using the Rapid Estimate of Adult Literacy in Medicine (REALM) questionnaire,^15^ as well as the usage frequency of how often participants use voice assistants (Table 6).

### Apparatus

Instead of speaking live into each voice assistant, participants’ voices were recorded in order to use the same audio clips to play back to each device during analyses. Participant voice recordings were captured via an Audio-Technica AT2020 Cardioid Condenser Microphone and QuickTime software on a MacBook Air laptop computer. During analyses, voice recordings were played back from the laptop using a Jabra Speak 410 speaker that was placed directly adjacent to the microphones of the voice assistant devices.

Alexa (Amazon), Google Assistant (Google), and Siri (Apple) were selected as voice assistants since they were the most popular and widely used consumer options at the time of the study, which took place in December 2018 to January 2019. Alexa was analyzed using a first-generation Amazon Echo smart speaker; Google Assistant was analyzed using a Samsung Galaxy S8 smartphone; Siri was analyzed using an iPhone 6 smartphone. All hardware devices were updated with their latest software available, and device language was set for English (Canada). All three assistants were connected to the internet using the Klick Inc. network.

### Procedure

After informed consent, participants completed the REALM questionnaire and reported their demographic information. From this point onward, all participant responses were audio-recorded to play back to each voice assistant in a controlled manner. To assess the baseline comprehension performance of the voice assistants, each participant asked three calibration questions (“*What day is it today?*”, “*What is 10* + *10?*”, “*What is the capital of France?*”). Calibration questions were used primarily to test the speech recognition quality of the devices and to make sure the system was working properly for each participant’s voice.

Participants were then instructed to read a list of all the brand name medications presented to them (Supplementary Table 2), followed by a list of all the generic name medications (Supplementary Table 1) presented to them. Brand names were always read before generic names since they were assumed to be easier to read and were expected to “warm up” the participants for pronouncing complex medications. The presentation order of the medication names within each list was randomized for each participant. For each medication name, participants were instructed to state the phrase “*Tell me about…*” followed by the drug name on the list (e.g., “*Tell me about acetaminophen*”). Participants were asked to pronounce each name as best they could, and if they felt that they mispronounced a word, they were welcome to say it again. No feedback was given to participants after each medication name as to whether they correctly pronounced the word or not. No maximum amount of pronunciation attempts were implemented, but the great majority of participants announced each name just once, and usually no more than two or three times. Only their best recording for each name was used for analyses.

### Data analysis

All voice recordings were analyzed between mid-December 2018 and mid-January 2019, using the latest software updates of all the voice assistants before analyses. No manual updates were performed on the devices during the analysis period. The relatively short time frame of the analysis was to minimize any potential improvements of the algorithms from each company’s technology.

Calibration questions from each participant were first played back to each device used to assess baseline comprehension performance of the voice assistants, as well as to adjust the volume of the audio-recording playback if needed. All 46 participants elicited 100% comprehension accuracy on all three voice assistants for each calibration question. In other words, all of the voice assistants yielded perfect accuracy on speech recognition from each participant for generic queries. Therefore, it could be inferred that any reduction in comprehension accuracy for each medication name would be based on the AI ability of the software to recognize the complexity of the drug name, and not due to the hardware used for recording and playback, or to the incomprehensibility of the participants’ voices. Additionally, calibration questions were purposely designed to be relatively easy and simple to pronounce using common words and phrases to assess the baseline measurement of comprehension performance of each voice assistant, which in this case, was intended to be 100% accuracy. Although medication names range in commonality and complexity from the calibration statements, the purpose of the current study was to test any detriment of speech recognition for drug names compared to the baseline performance of common voice assistant commands for everyday tasks.

Individual audio clips of each drug name (e.g., “*Tell me about [medication name]*”) from each participant were played back to each voice assistant. Although the reading ability of medication names by the participants was not directly being tested, their audio clips were scored by the authors using established norms^16,17^ as to whether the names were pronounced correctly or not. Each medication name pronunciation was scored in one of three ways:

i. *Incorrect*: the participant did not pronounce the word correctly at all (e.g., either by missing syllables, adding extra letters, or rearranging the phonetic pronunciation of each syllable).
ii. *Partially correct*: the participant pronounced each syllable of the word correctly, but placed a different emphasis on the wrong syllable (e.g., pronouncing alprazolam as “*al-pra-ZO-lam*” as opposed to the correct way of “*al-PRA-zo-lam*”).
iii. *Fully correct*: the participant pronounced the word correctly, including the proper enunciation of each syllable in the word.

Arguably, methods ii) and iii) of pronouncing the word are both “correct”. As an analogy, this is similar to the pronunciation of the word “tomato”, which may be commonly pronounced as “*tuh-MAY-toh*” in North America, but “*tuh-MAH-toh*” in the UK, and it would be likely that a restaurant chef would understand what a patron requests in their food order regardless of how they say the word or which country they are in. Similarly, a pharmacist or physician is likely to have a high probability of understanding a medication name regardless of whether a patient placed emphasis on different syllables of a drug name, as long as all the correct syllables were in the word. For statistical analyses, all of the participants’ voice recordings and pronunciations were used to playback to the voice assistants (Table 1). That is, incorrect pronunciations of words were not excluded as to represent real-world behaviors of speaking complex medications, as well as to examine the AI capabilities of the software (see results for further analyses); voice assistants were sometimes able to correctly recognize a word, even when incorrectly pronounced by the participant.

After the audio clips of each medication name were played back to the voice assistants, the devices’ responses were also scored in one of three ways:

i. *No response*: the voice assistant did not recognize any form of the query (e.g., by stating, “*Sorry, I don’t know that*”), and did not display any results.
ii. *Misinterpreted*: the voice assistant comprehended a different word or sentence and responded inaccurately with an irrelevant answer.
iii. *Accurate*: the voice assistant comprehended the medication name accurately, and provided a relevant response based on the drug.

Only response type iii) was scored as a valid response and was the main dependent variable of comprehension accuracy. Voice assistant responses were not scored on the quality or usefulness of information received or where the AI system sourced its information (e.g., Wikipedia, WebMD, World Health Organization, etc.). The only variable of interest was whether the voice assistant accurately recognized the medication name when being orally spoken by a variety of individuals. No feedback was given to the voice assistants after each response, so as not to potentially alter their algorithms or response patterns.

No statistical comparisons were conducted on misinterpreted responses (see type ii above) or no responses (see type i above), i.e., error rates, largely because Alexa was the only voice assistant who yielded “no response” scores (see Tables 2 and 3). By contrast, Google Assistant and Siri would always reveal a misinterpreted response when making a speech recognition error or faced with an incomprehensible medication name. Therefore, the misinterpreted response rates of Google Assistant and Siri would be the exact inverse of the accurate response rates, and were not reported due to redundancy.

Comprehension accuracy rates (i.e., accurate responses) were analyzed with a 2 (name type: brand medication, generic medication) x 3 (voice assistant: Alexa, Google Assistant, Siri) repeated measures analysis of variance (ANOVA), with participant accent (Canadian accent, foreign accent) as a between-subjects factor. Post-hoc *t*-tests (two-sided) were used to analyze differences in comprehension accuracy across voice assistants. Analyses revealed no significant effects of participant age or gender on comprehension accuracy across voice assistants.

### Reporting Summary

Further information on research design is available in the Nature Research Reporting Summary linked to this article.

## Supplementary information

Reporting Summary

Supplementary Information.

## Data Availability

The data that support the findings of this study are available from the corresponding author upon reasonable request.

## Supplementary information

**Supplementary information** accompanies the paper on the *npj Digital Medicine* website (10.1038/s41746-019-0133-x).

## References

[1] Hoy MB (2018). Alexa, Siri, Cortana, and more: an introduction to voice assistants. Med. Ref. Serv. Q..

[2] Pew Research Center. *Fact-tank* https://www.pewresearch.org/fact-tank/2017/12/12/nearly-half-of-americans-use-digital-voice-assistants-mostly-on-their-smartphones/ (2017).

[3] Jeffs, M. *OK Google, Siri, Alexa, Cortana; Can You Tell Me Some Stats On Voice Search?* https://edit.co.uk/blog/google-voice-search-stats-growth-trends/ (2018).

[4] Chung AE, Griffin AC, Selezneva D, Gotz D (2018). Health and fitness apps for hands-free voice-activated assistants: content analysis. JMIR Mhealth Uhealth.

[5] Miner AS (2016). Smartphone-based conversational agents and responses to questions about mental health, interpersonal violence, and physical health. JAMA Intern. Med..

[6] Bickmore TW (2018). Patient and consumer safety risks when using conversational assistants for medical information: an observational study of Siri, Alexa, and Google Assistant. JMIR.

[7] Boyd M, Wilson N (2018). Just ask Siri? A pilot study comparing smartphone digital assistants and laptop Google searches for smoking cessation advice. PLoS ONE.

[8] Fuentes AV, Pineda MD, Venkata KCN (2018). Comprehension of top 200 prescribed drugs in the us as a resource for pharmacy teaching, training and practice. Pharmacy.

[9] Statista. *Total number of Medical Prescriptions Dispensed in the U.S. from 2009 to 2016* https://www.statista.com/statistics/238702/us-total-medical-prescriptions-issued/ (2019).

[10] ClinCalc. *The Top 200 Drugs of 2019* https://clincalc.com/DrugStats/ (2019).

[11] Wired. *8 People Test Their Accents on Siri, Echo and Google Home* https://www.youtube.com/watch?v=gNx0huL9qsQ (2017).

[12] Stone Temple. *Rating the Smarts of the Digital Personal Assistants in 2018* https://www.stonetemple.com/digital-personal-assistants-study/ (accessed, 12 February 2019).

[13] Ho DK-h (2018). Voice-controlled virtual assistants for the older people with visual impairment. Eye.

[14] Zhou L (2018). Analysis of errors in dictated clinical documents assisted by speech recognition software and professional transcriptionists. JAMA Open.

[15] Davis TC (1993). Rapid estimate of adult literacy in medicine: a shortened screening instrument. Fam. Med..

[16] ClinCalc. *How to Pronounce the Top 250 Drugs* https://clincalc.com/PronounceTop200Drugs/ (2019).

[17] Li, V. *Top 200 Drugs Pronunciation (Generic Names/ Brand Names)* https://www.youtube.com/watch?v=K007kEeN6Gg (2016).

