# Supplementary material for: Do you understand the words that are comin outta my mouth? Voice assistant comprehension of medication names
Source: NPJ Digit Med. 2019 Jun 20;2:55. doi: 10.1038/s41746-019-0133-x (PMC6586879; doi:10.1038/s41746-019-0133-x)
Supplement: Supplementary file 2 — Supplementary Information. [file 41746_2019_133_MOESM2_ESM.docx]

| Supplementary Table 1. Generic Names of the Top 50 Most Dispensed Medications in the United States, in Alphabetical Order, with Relative Comprehension Accuracy for Each Name (N = 46) | | | |
| --- | --- | --- | --- |
| Generic Medications | Alexa Comprehension Accuracy Mean % | Google Assistant Comprehension Accuracy Mean % | Siri Comprehension Accuracy Mean % |
| 1. Acetaminophen | 59 | 80 | 72 |
| 1. Albuterol | 41 | 83 | 67 |
| 1. Alprazolam | 52 | 89 | 74 |
| 1. Amlodipine | 41 | 80 | 50 |
| 1. Amoxicillin | 80 | 98 | 83 |
| 1. Aspirin | 93 | 100 | 93 |
| 1. Atenolol | 37 | 80 | 57 |
| 1. Atorvastatin | 33 | 65 | 43 |
| 1. Azithromycin | 46 | 70 | 28 |
| 1. Bupropion | 24 | 61 | 33 |
| 1. Carvedilol | 43 | 76 | 41 |
| 1. Citalopram | 0 | 85 | 33 |
| 1. Clonazepam | 57 | 89 | 61 |
| 1. Clopidogrel | 15 | 59 | 24 |
| 1. Cyclobenzaprine | 74 | 96 | 83 |
| 1. Duloxetine | 15 | 78 | 39 |
| 1. Escitalopram | 24 | 50 | 4 |
| 1. Ethinylestradiol | 26 | 48 | 26 |
| 1. Fluoxetine | 33 | 89 | 59 |
| 1. Fluticasone | 22 | 65 | 37 |
| 1. Furosemide | 41 | 83 | 43 |
| 1. Gabapentin | 85 | 96 | 89 |
| 1. Glipizide | 43 | 78 | 50 |
| 1. Hydrochlorothiazide | 63 | 100 | 65 |
| 1. Hydrocodone | 96 | 100 | 85 |
| 1. Ibuprofen | 4 | 98 | 89 |
| 1. Insulin Glargine | 11 | 89 | 39 |
| 1. Levothyroxine | 7 | 80 | 15 |
| 1. Lisinopril | 11 | 78 | 39 |
| 1. Losartan | 46 | 83 | 20 |
| 1. Meloxicam | 70 | 89 | 39 |
| 1. Metformin | 59 | 87 | 43 |
| 1. Metoprolol | 17 | 87 | 7 |
| 1. Montelukast | 70 | 93 | 13 |
| 1. Omeprazole | 50 | 96 | 43 |
| 1. Oxycodone | 96 | 98 | 96 |
| 1. Pantoprazole | 24 | 93 | 67 |
| 1. Potassium Chloride | 100 | 100 | 74 |
| 1. Pravastatin | 46 | 96 | 28 |
| 1. Prednisone | 70 | 91 | 74 |
| 1. Ranitidine | 17 | 83 | 37 |
| 1. Rosuvastatin | 0 | 48 | 24 |
| 1. Sertraline | 39 | 87 | 50 |
| 1. Simvastatin | 48 | 100 | 30 |
| 1. Tamsulosin | 50 | 78 | 59 |
| 1. Tramadol | 89 | 100 | 91 |
| 1. Trazodone | 61 | 98 | 83 |
| 1. Venlafaxine | 54 | 76 | 52 |
| 1. Warfarin | 41 | 98 | 59 |
| 1. Zolpidem | 54 | 89 | 50 |

| Supplementary Table 2. Brand Names of the Top 50 Most Dispensed Medications in the United States, Ordered in Reference to the Alphabetical Sequence of Appendix A, with Relative Comprehension Accuracy for Each Name (N = 46) | | | |
| --- | --- | --- | --- |
| Generic Medications | Alexa Comprehension Accuracy Mean % | Google Assistant Comprehension Accuracy Mean % | Siri Comprehension Accuracy Mean % |
| 1. Tylenol | 98 | 98 | 93 |
| 1. Ventolin | 70 | 100 | 67 |
| 1. Xanax | 87 | 98 | 93 |
| 1. Norvasc | 70 | 91 | 72 |
| 1. Amoxil | 35 | 91 | 13 |
| 1. Bayer | 7 | 83 | 17 |
| 1. Tenormin | 30 | 80 | 20 |
| 1. Lipitor | 89 | 100 | 87 |
| 1. Zithromax | 52 | 98 | 67 |
| 1. Wellbutrin | 80 | 96 | 7 |
| 1. Coreg | 59 | 96 | 9 |
| 1. Celexa | 87 | 100 | 89 |
| 1. Klonopin | 74 | 100 | 72 |
| 1. Plavix | 37 | 100 | 67 |
| 1. Flexeril | 70 | 96 | 89 |
| 1. Cymbalta | 83 | 96 | 83 |
| 1. Lexapro | 59 | 100 | 93 |
| 1. Ortho Tri-Cyclen | 0 | 98 | 93 |
| 1. Prozac | 98 | 100 | 96 |
| 1. Flonase | 78 | 96 | 83 |
| 1. Lasix | 63 | 80 | 43 |
| 1. Neurontin | 57 | 93 | 63 |
| 1. Glucotrol | 43 | 80 | 43 |
| 1. Microzide | 33 | 48 | 54 |
| 1. Vicodin | 74 | 96 | 33 |
| 1. Advil | 2 | 100 | 59 |
| 1. Lantus | 39 | 100 | 11 |
| 1. Levaquin | 76 | 100 | 80 |
| 1. Prinivil | 11 | 93 | 33 |
| 1. Cozaar | 2 | 96 | 4 |
| 1. Mobic | 85 | 100 | 67 |
| 1. Glucophage | 74 | 98 | 85 |
| 1. Lopressor | 78 | 100 | 57 |
| 1. Singulair | 67 | 100 | 52 |
| 1. Prilosec | 59 | 87 | 46 |
| 1. Oxycontin | 98 | 100 | 72 |
| 1. Protonix | 78 | 96 | 83 |
| 1. K-Dur | 0 | 0 | 0 |
| 1. Pravachol | 20 | 89 | 24 |
| 1. Deltasone | 11 | 91 | 33 |
| 1. Zantac | 89 | 100 | 87 |
| 1. Crestor | 70 | 100 | 65 |
| 1. Zoloft | 80 | 100 | 91 |
| 1. Zocor | 78 | 96 | 70 |
| 1. Flomax | 93 | 100 | 98 |
| 1. Ultram | 4 | 100 | 20 |
| 1. Desyrel | 11 | 41 | 22 |
| 1. Effexor | 7 | 91 | 63 |
| 1. Coumadin | 43 | 98 | 59 |
| 1. Ambien | 24 | 100 | 100 |
